# Is sleep bruxism in obstructive sleep apnea only an oral health related problem?

**DOI:** 10.1186/s12903-024-04351-1

**Published:** 2024-05-14

**Authors:** J. Sambale, U. Koehler, R. Conradt, K. Kesper, W. Cassel, M. Degerli, C. Viniol, H. M. Korbmacher-Steiner

**Affiliations:** 1 Department of Orthodontics, Clinic of Dentistry, Philipps-University Marburg, Georg-Voigt-Str. 3, 35041 Marburg, Germany; 2 Departement of Pneumology, Philipps-University Marburg, Marburg, Germany; 3 Faculty of Health Sciences at the University of Applied Sciences, Gießen, Germany

**Keywords:** Obstructive sleep apnea, Sleep bruxism, Clinical muscle symptoms, Temporomandibular disorder, Electromyographic muscle tone, Muscle tone, Polysomnography

## Abstract

**Background:**

The etiology of sleep bruxism in obstructive sleep apnea (OSA) patients is not yet fully clarified. This prospective clinical study aimed to investigate the connection between probable sleep bruxism, electromyographic muscle tone, and respiratory sleep patterns recorded during polysomnography.

**Methods:**

106 patients with OSA (74 males, 31 females, mean age: 56.1 ± 11.4 years) were divided into two groups (sleep bruxism: SB; no sleep bruxism: NSB). Probable SB were based on the AASM criteria: self-report of clenching/grinding, orofacial symptoms upon awakening, abnormal tooth wear and hypertrophy of the masseter muscle. Both groups underwent clinical examination for painful muscle symptoms aligned with Temporomandibular Disorders Diagnostic Criteria (DC/TMD), such as myalgia, myofascial pain, and headache attributed to temporomandibular disorder. Additionally, non-complaint positive muscle palpation and orofacial-related limitations (Jaw Functional Limited Scale-20: JFLS-20) were assessed. A one-night polysomnography with electromyographic masseter muscle tone (EMG) measurement was performed. Descriptive data, inter-group comparisons and multivariate logistic regression were calculated.

**Results:**

OSA patients had a 37.1% prevalence of SB. EMG muscle tone (N1-N3, REM; *P* = 0.001) and the number of hypopneas (*P* = 0.042) were significantly higher in the sleep bruxism group. While measures like apnea–hypopnea-index (AHI), respiratory-disturbance-index (RDI), apnea index (AI), hypopnea-index (HI), number of arousals, and heart rate (1/min) were elevated in sleep bruxers, the differences were not statistically significant. There was no difference in sleep efficiency (SE; *P* = 0.403). Non-complaint masseter muscle palpation (61.5%; *P* = 0.015) and myalgia (41%; *P* = 0.010) were significant higher in SB patients. Multivariate logistic regression showed a significant contribution of EMG muscle tone and JFLS-20 to bruxism risk.

**Conclusion:**

Increased EMG muscle tone and orofacial limitations can predict sleep bruxism in OSA patients. Besides, SB patients suffer more from sleep disorder breathing. Thus, sleep bruxism seems to be not only an oral health related problem in obstructive apnea. Consequently, interdisciplinary interventions are crucial for effectively treating these patients.

**Trial registration:**

The study was approved by the Ethics Committee of Philipps-University Marburg (reg. no. 13/22—2022) and registered at the “German Clinical Trial Register, DRKS” (DRKS0002959).

## Background

There appears to be a frequent temporal association between obstructive sleep apnea (OSA) and sleep bruxism (SB) [1–3]. OSA is a chronic sleep disorder characterized by repeated episodes of partial or complete obstruction of the pharyngeal airway during sleep [4]. Many studies have reported that OSA is highly prevalent in the general population [5]. Anatomical factors such as craniofacial anomalies and obesity are recognized as a major risk factor for OSA [6, 7]. But with increasing age the importance of obesity decreases since a higher prevalence of OSA has been reported among patients with normal weight. The reason is a transformation of muscle fibers in the upper airway due to the effects of chronic intermittent hypoxemia, low-grade inflammation and increased sympathetic tone resulting in reduced muscle mass and strength [8]. Despite the reduced muscle mass with increased age prevalence of SB among OSA patients with masseter muscle activation ranges from 26%-54% and is therefore much higher than in healthy subjects with a known prevalence of 12.8 ± 3.1% [3, 9–12]. SB is a multifactorial oral condition and is not a movement or sleep-related disorder in healthy individuals [2]. While in these healthy subjects SB is often associated with anxiety, psychologic stress, habitual use of exogenous substances (eg, smoking and alcohol) and genetic predisposition, the etiology of sleep bruxism in obstructive sleep apnea patients is still not fully clarified. In dentistry, SB has long been interpreted as a clinical risk factor on tooth wear, damage and loose [13], higher failure in prostheses [14, 15], higher complication rate in dental implants [15], morning fatigue and pain in the masticatory muscles [16] and association with temporomandibular disorders (TMD)[17–22]. In addition, some authors also reported that SB seemed to have a negative impact on sleep quality and quality of life [23–25], while others reported that sleep quality remained unchanged [26]. Beside these reports of the negative aspects of SB on oral health and sleep quality, masseter muscle activation has been proposed as a possible protective factor to protrude the mandible and increase airway patency to reduce the severity of OSA [27].

There are documented positive associations between rhythmic masticatory muscle activity (RMMA) in jaw elevator muscles and obstructive apnea. Tan et al. [9] found in a retrospective investigation an association between the apnea–hypopnea index and SB. They reported a significantly higher respiratory arousal index and oxygen desaturation index in SB patients. Hosoya et al. [26] demonstrated positive correlations between SB events and obstructive apnea, desaturation, and microarousal event indices, suggesting that OSA might be a high-risk factor for SB. However, there were also heterogenous results regarding SB activity over more than one recorded night [28]. Some studies concluded that the RMMA activities remains constant over time, while others reported a high variability in RMMA activity [28–31]. To date, there hasn’t been any study that has quantified the average electromyographic activity in masticatory muscles during polysomnography (PSG) in OSA patients probable to experience sleep bruxism, and those without bruxism. Raphael et al. [32] compared background EMG activity during non-SB event periods in TMD patients and in controls. The results showed that background EMG levels are significantly elevated in myofascial TMD patients compared to controls. Multiple researchers have highlighted a strong connection between sleep bruxism (SB) and Temporomandibular Disorders (TMD) [22, 33]. Manfredini et al. [34] underscored the significance of reassessing the classification of sleep bruxism diagnoses into possible, probable, or definite categories. He proposed a reconsideration of this approach within the broader construct of bruxism, recognizing it as an umbrella term encompassing a spectrum of diverse muscle activities.

Therefore, this study aimed to examine mean EMG muscle tone without removing phasic SB events. The objectives of this study were as follows:

1. To compare EMG muscle tone between sleep bruxers and non-sleep bruxers.
2. To assess a possible association to sleep macrostructure and respiratory variables between sleep bruxers and non-sleep bruxers.
3. To assess the influence of muscle tone and obstructive apnea.
4. To assess the prevalence of clinical painful and non-painful muscle symptoms in probable sleep bruxers.

We hypothesized that SB and muscle tone could influence obstructive apnea in a positive way to maintain breathing patency.

## Material and methods

### Subject characteristics and study design

The study was approved by the Ethics Committee of Philipps-University Marburg (reg. no. 13/22—2022) and registered at the “German Clinical Trial Register, DRKS” (DRKS0002959). Inclusion criteria were OSA with an apnea–hypopnea index (AHI) ≥ 10 events h^−1^ and a minimum of 4 h total sleep time (TST) confirmed by the stationary PSG, age > 18 years, permanent dentition, and number of teeth > 20. The exclusion criteria were lack of patient’s willingness to sign an informed consent form, age < 18 years, any neurological, psychiatric or sleep disorders other than sleep apnea other than obstructive sleep apnea, neuropathic pain with medication intake and impairment of muscle and respiratory function and psychoactive medication intake with risk of jaw muscle and/or limb activity; and removable dentures. Based on anamnestic and clinical assessment of probable SB, patients were allocated to either a sleep bruxism group (SB) or no sleep bruxism group (NSB). In total 106 patients (73 men, 33 women) with mean age of 55.9 ± 11.9 years were recruited.

### Anamnestic and clinical assessment

The anamnestic and clinical assessment were performed by an orthodontist specialized in dental sleep medicine (JS). Sleep bruxism diagnosis was based on the AASM criteria [4]. Patients with probable sleep bruxism reported at least one anamnestic symptom: awareness of painful, tiredness, stiffness and limited or painful jaw opening upon awakening, morning headache and/or tooth grinding noises occurring at least 3–5 nights per week over 6 months reported by a sleep partner and at least one clinical sign: tooth wear according to Wetselaar & Lobbezoo [35] (grade > 1) and hypertrophy of the masseter muscle. The criteria used to categorize individuals into either the SB or NSB group are detailed in Table 1.

**Table 1:** Protocol for group assignment

Diagnostic	Diagnostic result			
Anamnestic symptoms^a^	No	Yes	No	Yes
Clinical symptoms^a^	No	No	Yes	Yes
Group assignment	NSB	NSB^b^	NSB^c^	SB

^a^At least one anamnestic and one clinical symptom based on AASM criteria [1]

^b^Patient overestimated anamnestic symptoms

^c^Suspected wake bruxism

Furthermore, each patient filled in a DC/TMD symptomatic questionnaire and Jaw Functional Limited Scale (JFLS-20) [36, 37]. Both groups were clinically examined using the diagnostic criteria for temporomandibular disorders (DC/TMD) for painful muscle symptoms [37]. Diagnosis for painful muscle symptoms were myalgia, myofascial pain and headache attributed to TMD. Furthermore, non-complaint muscle symptoms were examined, which were defined as positive palpation diagnosis of masseter and/or temporalis muscles without daily impairment.

### Polysomnographic sleep recording and OSA scoring

Each patient underwent a polysomnography (PSG) recording (type Sonata, Löwenstein Medicals, Bad Ems, Germany) for a single night. According to the AASM standards [38] the recording variables included surface electrodes at the following locations: electroencephalograms (EEG) at C3-M2, C4-M1, O1-M2 and O2-M1, an electrocardiogram (ECG), bilateral electrooculogram (EOG) and electromyograms (EMG) of the submental and tibialis muscle. Additional, EMG electrodes of bilateral masseter muscle were used. Calibration of the EMG electrodes was performed by three times maximal clenching prior to “lights-off”. Airflow was monitored with oro-nasal thermistors and nasal prongs, chest and abdominal inductive plethysmography (two channels) was measured and SpO_2_ was measured with pulse oximetry (one channel). Sleep stages and respiratory events were scored visually according to current AASM criteria [38]. The following variables were calculated by Miniscreen SW (Löwenstein Medical, Bad, Ems, Germany): percentage of time spent in sleep stages N1, N2, N3, and REM (time spent in each sleep stage/total sleep time*100%), total sleep time (TST: minutes asleep in bed after “ lights off”, considering only nighttime sleep), sleep efficiency (SE: total sleep time/total time in bed * 100%), apnea hypopnea index per hour sleep (AHI/h), respiratory disturbance index per hour sleep (RDI/h), apnea index and hypopnea index per hour sleep (AI/h, HI/h), oxygen desaturation index per hour sleep (ODI/h), mean desaturation per total sleep time (in % and in sec.) total number of hypopnea and arousals per total sleep time, snoring index per hour sleep (SI/h), average pulse (1/min) and average heart rate (1/min) per total sleep time.

### Assessment of electromyographic muscle tone

Surface electrodes for EMG were affixed by a sleep technician to the primary muscle belly of the left and right masseter muscles. The sleep technician was blinded to the clinical diagnosis. The accurate positioning on the cheek was ascertained when the patient applied substantial occlusal force to their dentition. Before starting the polysomnographic sleep recording the EMG electrodes were calibrated by three times maximum voluntary contraction. The software development for muscle tone analysis was performed by a computer scientist (KK) who was not involved in the recruitment process and blinded to the clinical diagnosis.

Masseter EMG tone was determined by an automated computer analysis [39] and evaluated specifically for the different sleep stages. Masseter EMG signals were filtered with a band-pass (10—100 Hz) and a 50 Hz notch filter to remove interference by the mains. When encountering ECG interference, an adaptive ECG artifact filter was employed to eliminate artifacts [40]. In these instances, the polysomnographically recorded ECG signal was also necessary.

**Fig. 1:**
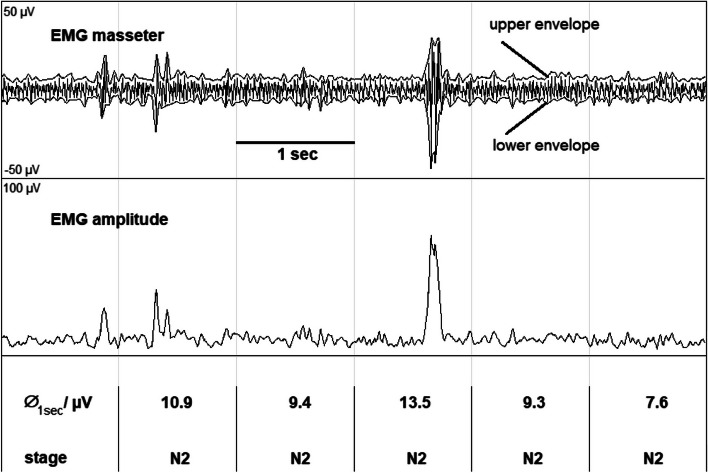
Upper and lower envelopes of the masseter EMG were subtracted to calculate the EMG amplitude. EMG tone values (∅_1 s_) were calculated by averaging the EMG amplitude within each 1-s interval. Mean muscle tone values were calculated subsequently for each sleep stages (N1-N3, REM)

The automatic analysis calculated the muscle tone via following steps: First, upper and lower envelopes of the EMG were calculated and averaged over 5 individual samples (corresponding to 25 ms for an EMG sampled at 200 Hz). The envelopes were subtracted to get the EMG amplitude shown in Fig. 1.

Muscle tone values were calculated as the mean amplitude within each 1-s interval and listed with the corresponding sleep stages. These values represented the data basis for the calculation of the stage-specific statistical parameters (mean, standard deviation, number) of the masseter EMG tone.

Periods with phasic EMG activations were not excluded by the automatic analysis because the overall duration of phasic events was normally very short compared to that of tonic EMG.

### Statistical analysis

Statistical analyses were performed using IBM SPSS Statistics (Version 29.0, IBM Corp.). Descriptive statistics were performed for the demographic characteristics (age, gender, BMI) of patients. Frequencies were expressed as percentage values and frequency rates were compared using Fisher’s exact test. For the polysomnographic respiratory variables and EMG tonus measurements the Kolmogorov–Smirnov test was performed on all variables to test for normality of distribution. The data were summarized with means and standard deviation (SD) and if there was not a normal distribution with medians and interquartile range (IQR). Mann–Whitney *U* tests and independent sample t tests were performed for intergroup comparisons of respiratory variables and EMG tonus measurements. Kruskal–Wallis-H-test was performed to evaluate differences in the EMG amplitudes for the three apnea severity groups (light: AHI < 15, moderate: AHI ≥ 15 < 30, severe: AHI ≥ 30). Pearson correlation coefficient (PCC) was calculated to measure linear correlations between mean EMG tonus in TST (mean of measurements of right and left electrode for all EMG tonus measurements in TST) and respiratory variables, such as between mean EMG tonus in TST and clinical variables (JFLS-20, tooth wear). Anamnestic variables and mean EMG tonus measurements in TST were correlated using a logistic regression model with having or not having SB as the dichotomous outcome variable. Statistical significance was set at *P* < 0.05.

## Results

In total data of 105 patients could be statistically analyzed. One patient was excluded because of PSG failure. Based on anamnestic and clinical symptoms 39 subjects (25 males, 14 females) with mean age of 54.4 ± 12.6 years and mean BMI of 31.4 ± 5.8 kg/m^2^ were allocated to SB group. 66 subjects (49 males, 17 females) with mean age of 57.7 ± 10.5 years and a mean BMI of 30.9 ± 6.9 kg/m^2^ were allocated to NSB group. The Mann–Whitney *U* test showed no significant difference between the two groups in age (*P* = 0.114) and BMI (*P* = 0.385).

### EMG muscle tone data

**Fig. 2:**
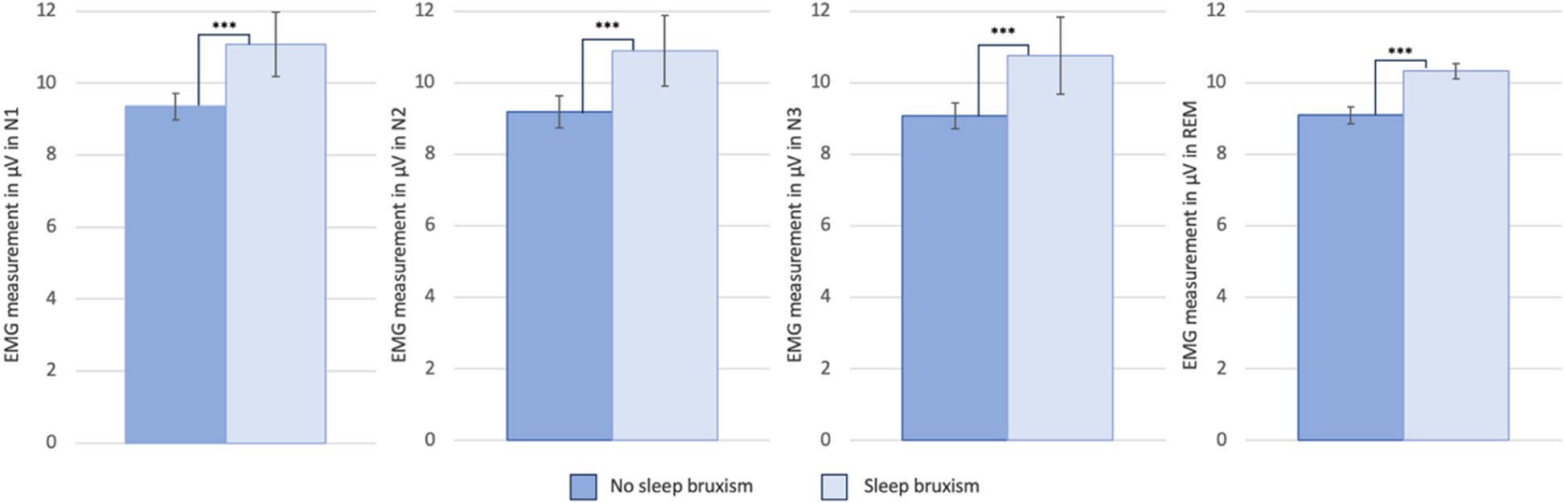
Electromyographic mean muscle tone and standard deviation (SD) in µV in sleep stages N1, N2, N3 and REM. Independent samples t test. ***P < 0.001

Inter-group comparison of electromyographic muscle tone is shown in Fig. 2. In both groups, mean muscle tone was lowest in sleep stage REM (SB: 10.17 ± 0.65 µV; NSB: 8,98 ± 1.00 µV) and highest in sleep stage N1 (SB: 11.04 ± 0.78 µV; NSB: 9.33 ± 1.21 µV). In all sleep stages (N1, N2, N3, REM), mean EMG muscle tone was significantly higher in the SB group (*P* < 0.001).

### Polysomnographic respiratory variables

**Fig. 3:**
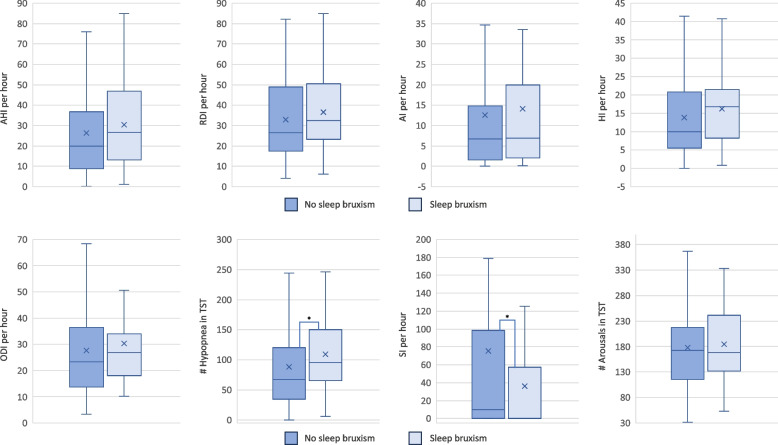
Distribution of the respiratory sleep variables AHI/h, RDI/h, AI/h, HI/h, ODI/h, hypopnea (n in TST), snoring index per hour (SI/h) and number of arousals in TST for the SB group and NSB group. Box plots are shown. The Mann–Whitney *U* test was used for pairwise comparisons. *P < 0.05

**Fig. 4:**
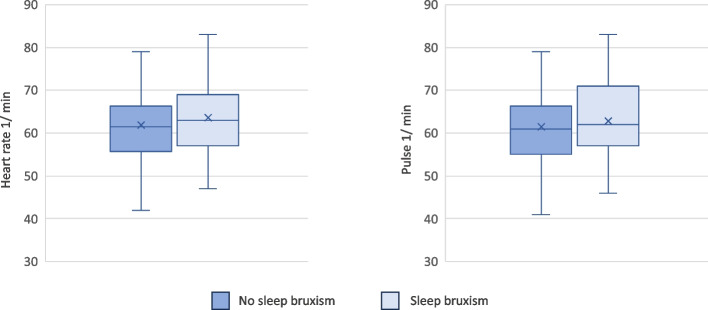
Box plots of heart rate (1/min.) and pulse (1/min) of the two groups are shown. Mann–Whitney *U* test was used for pairwise comparisons

Polysomnographic differences in respiratory parameters are shown in Figs. 3 and 4. No significant differences were found for the variables AHI/h (*P* = 0.220), RDI/h (*P* = 0.158), AI/h (*P* = 0.529), HI/h (*P* = 0.114), ODI/h (*P* = 0.284), number of apnea in TST (*P* = 0.387), number of arousals in TST (*P* = 0.698) (Fig. 3), mean heart rate in TST (1/min) (*P* = 0.334) and mean pulse in TST (1/min) (*P* = 0.403) (Fig. 4) among the two groups. However, the number of hypopneas in TST were significantly higher in the SB group (*P* = 0.042) and the snoring index (SI/h) showed a significantly higher value in the NSB group (*P* = 0.033) (Fig. 2).

### EMG muscle tone and respiratory parameters

The median EMG values for the three apnea severity groups were 9.22 µV (AHI/h < 15), 9.46 µV (AHI/h ≥ 15—< 30), and 9.67 µV (AHI/h ≥ 30) (Kruskal–Wallis-H-test, *P* = 0.114). The respiratory variables AHI/h, AI/h, RDI/h, ODI/h and mean heart rate (1/min) in TST correlated positively with EMG muscle tone, while SI/h correlated negatively with EMG muscle tone. A significant positive correlation was noted between EMG muscle tone and mean desaturation (in %; *P* = 0.031), while a significant negative correlation was noted between EMG muscle tone and mean desaturation duration (in sec.; *P* = 0.001) (Table 2).

**Table 2:** Pearson correlation between mean EMG muscle tone in µV in TST and respiratory variables

	**Mean EMG muscle tone in µV in TST in OSA patients**	
**Respiratory variables**	*Pearson correlation*	*P* value
AHI/h	0.150	0.194
AI/h	0.052	0.652
HI/h	0.219	0.056
RDI/h	0.160	0.166
ODI/h	0.126	0.274
SI/h	-0.022	0.850
Desaturation (mean duration in sec.)	-0.376	0.001**
Desaturation (mean in %)	0.246	0.031*
Mean heart rate (1/min) in TST	0.035	0.762

^*^P < 0.05

^**^P < 0.01

### Sleep stages and sleep macrostructure

Total sleep time (TST; *P* = 0.326), sleep efficiency (SE; *P* = 0.403) and sleep stages (N1: *P* = 0.793, N2: *P* = 0.776, N3: *P* = 0.535, REM: *P* = 0.738) were not statistically different between both groups (Table 3).

**Table 3:** Comparison of sleep stages (N1-N3, REM), TST and SE

	**SB**				**NSB**				
**PSG variables**	**Mean**	**SD**	**Median**	**IQR**	**Mean**	**SD**	**Median**	**IQR**	***P* value**
N1 (in %)^a^	6.49	6.72	5.30	2.70–8.40	6.39	4.90	5.10	3.60–7.58	0.793
N2 (in %)^a^	50.78	9.86	49.20	45.20–56.00	51.23	12.19	52.15	42.03–59.35	0.776
N3 (in %)^a^	25.23	10.95	26.60	17.00–33.00	24.88	10.88	23.00	16.60–31.88	0.535
REM (in %)^a^	17.48	5.88	18.00	13.90–22.10	17.50	7.74	17.00	11.95–22.38	0.738
TST (in min.)^a^	391.75	65.56	392.82	340.54–400.36	378.97	66.16	380.12	329.22–391.75	0.326
SE (in %)^a^	80.23	10.30	79.96	76.79–83.17	78.21	11.63	77.94	75.31–80.38	0.403

N1-N3, REM: time spent in each sleep stage/total sleep time*100%; TST: minutes asleep in bed after “lights off,” considering only nighttime sleep; SE: total sleep time/total time in bed * 100%

*SD* Standard deviation, *IQR* Interquartile range

^a^Mann-Whitney *U* test

### Predictability of sleep bruxism

Anamnestic data (age), oral health-related symptoms (JFLS-20) and mean EMG muscle tone were analyzed in a multivariate logistic regression model (Table 4). The odds ratio decreased by 4% for every age increase of one year without significance. In contrast, the odds of experiencing SB increased significantly by 8% for every score in JFLS-20 and by 2300% for EMG muscle tone increase of 1 µV.

**Table 4:** Multivariate logistic regression: predictability for sleep bruxism in OSA patient

Predictability factors	OR	95% CI	*P* value
Age	0.961	0.903–1.023	0.212
JFLS-20	1.080	1.013–1.151	0.019*
Mean EMG muscle tone in TST	23.697	5.084–110.448	< 0.001***

*OR* Odds ratio of age, orofacial symptoms (JFLS-20) and mean EMG muscle tone (mean of right and left EMG muscle tone in TST); *CI* Confidence interval

^*^P < 0.05

^***^P < 0.001

### Prevalence of anamnestic and clinical signs and symptoms

The distribution of positive anamnestic symptoms upon awakening, painful DC/TMD diagnosis and non-complaint positive muscle palpation are shown in Table 5. Statistically significant higher prevalence of all anamnestic and clinical muscle symptoms was found in the SB group. The prevalence of clenching and grinding was 64.0% in the SB group and 4.5% in the NSB group (*P* < 0.001). The SB group most often complained about morning jaw muscle pain (51.3%, *P* < 0.001). Positive non-complaint masseter palpation was the highest prevalence in both groups and intergroup comparison was statistically different (*P* = 0.015). Painful muscle DC/TMD diagnosis was detected with a statistically significant higher prevalence in SB group (61.5%) than in NSB group (21.2%) (*P* < 0.001). Regarding unspecific chewing and orofacial limitations, the SB group had significantly higher values in the JFLS-20 questionnaire than the NSB group (*P* = 0.002). Severity of tooth wear evaluation is shown in Table 6. Both groups showed a statistically significant difference in moderate (*P* = 0.010), severe (*P* < 0.001) and extreme tooth wear (*P* = 0.009).

**Table 5:** Prevalence of anamnestic and clinical muscle symptoms with intergroup comparison

Group assignment (%)	SB (37.1%)	NSB (62.9%)	Intergroup comparison (*P* value)
**Anamnestic symptoms (%)**			
Self-report of clenching and grinding^a^	64%	4.5%	< 0.001***
Morning jaw muscle pain^a^	51.3%	3.0%	< 0.001***
Morning jaw muscle tiredness^a^	38.5%	3.0%	< 0.001***
Morning jaw muscle stiffness^a^	38.2%	2.1%	< 0.001***
Morning headache^a^	46.2%	22.7%	0.017*
Morning limited jaw opening^a^	23.0%	3.0%	0.008**
**Clinical muscle symptoms (%)**			
Myofascial pain^a^	25.6%	7.6%	0.012*
Headache attributed to TMD^a^	17.9%	4.5%	0.037*
Positive masseter palpation^a^	61.5%	36.4%	0.015*
Positive temporalis palpation^a^	33.3%	3.0%	0.012*

^a^Fisher’s exact test

^*^P < 0.05

^**^P < 0.01

^***^P < 0.001

**Table 6:** Severity of tooth wear evaluation (grade 0 – 4) according to Wetselaar & Lobbezoo [35]

**Severity of tooth wear per number of teeth (in n)**	**SB**		**NSB**		
	**Mean**	**SD**	**Mean**	**SD**	***P* value**
No visible tooth wear (grade 0)^a^	2.79	4.14	6.39	5.33	/
Mild tooth wear (grade 1)^a^	13.69	5.85	14.33	4.80	0.616
Moderate tooth wear (grade 2)^a^	7.49	3.67	5.48	4.23	0.010*
Severe tooth wear (grade 3)^a^	2.46	2.65	0.55	1.13	< 0.001***
Extreme tooth wear (grade 4)^a^	0.46	1.07	0.14	1.00	0.009**

*SD* Standard deviation

^a^Independent samples t test

^*^ *P* < 0.05

^**^ *P* < 0.01

^***^ *P* < 0.001

### Correlation between EMG muscle tone and clinical signs

Correlation between EMG muscle tone and clinical signs is shown in Table 7. EMG muscle tone correlated significantly with JFLS-20 and severe tooth wear (*P* = 0.023), while there was a non-significant correlation with mild, moderate and extreme tooth wear.

**Table 7:** Pearson correlation: mean EMG muscle tone, clinical signs (tooth wear) and orofacial symptoms (JFLS-20)

	**Mean EMG muscle tone in µV in TST in OSA patients**	
**Clinical symptoms and signs**	*Pearson correlation*	*P* value
JFLS-20	0.235	0.046*
Mild tooth wear (grade 1)	0.027	0.815
Moderate tooth wear (grade 2)	0.110	0.340
Severe tooth wear (grade 3)	0.259	0.023*
Extreme tooth wear (grade 4)	0.094	0.414

^*^P < 0.05

## Discussion

This prospective clinical study explored the oral health indicators and symptoms associated with probable sleep bruxism among OSA patients. To the best of our knowledge this study represents the first attempt to evaluate the correlation between mean EMG muscle tone and obstructive apnea concerning both sleep and respiratory parameters. Sleep bruxism occurred in more than one-third of OSA patients with a prevalence of 37.1% in this study sample. The prevalence is comparable with the results of the retrospective polysomnographic investigation of Tan et al. [9]. They diagnosed SB with a prevalence of 33.3%, while other studies reported even higher prevalence [11, 41]. In contrast, sleep bruxism in healthy subjects has been reported in 12.8 ± 3.1% only [3, 9–12].

A group of bruxism experts proposed an international diagnostic grading system, which suggests three bruxism categories: Possible SB (based on positive self-report only), probable SB (based on a positive clinical inspection, with or without a positive self-report), and definite SB (based on a positive instrumental assessment, with or without a positive self-report and/or a positive clinical inspection) [2]. When identifying definite sleep bruxism it’s essential to consider that rhythmic masticatory muscle activity (RMMA) can fluctuate from night to night [28]. In daily dental practice it is necessary to detect patients with probable sleep bruxism and to find a standardization in clinical examination. In this study a standardized clinical examination procedure was used by a trained orthodontist (JS) to detect patients with probable sleep bruxism.

The average EMG muscle tone recorded across all sleep stages indicated the lowest levels during REM sleep, where muscle tone typically decreases significantly, and the highest levels during N1 sleep. However, there existed a statistically notable distinction, with elevated values across all sleep stages among patients with sleep bruxism. Furthermore, increased muscle tone showed an increase in orofacial symptoms (JFLS-20) such as increased tooth wear. To date, our study represents the initial attempt to measure the average EMG muscle tone in a substantial sample size of OSA patients, both with and without probable sleep bruxism. Raphael et al. [32] investigated masticatory muscle sleep background EMG in myofascial TMD patients. They excluded phasic EMG activities. In our study phasic RMMA events were not excluded, and muscle tone values were calculated as the mean amplitude within each 1-s interval and listed with the corresponding sleep stage in OSA patients with probable sleep bruxism. The multivariate logistic regression assessed the relationship between sleep bruxism in OSA patients and EMG muscle tone. The increased average EMG muscle tone emerged as a significant predictor of sleep bruxism.

Furthermore, sleep bruxism patients exhibited a higher frequency of respiratory events. An increased number of complete upper airway collapses were indicated by the AHI and AI. Sleep bruxism patients also showed a significantly higher number of incomplete obstructions, in terms of hypopneas, during sleep.

Regarding apnea severity groups muscle tone were highest in severe apnea (AHI ≥ 30), though it did not reach statistical significance; however, increasing the sample size could potentially yield significant results. In contrast, snoring (as in SI) yielded different results between bruxism and non-bruxism patients. Both groups showed snoring, but the snoring index was higher in the non-sleep bruxism group and with increased muscle tone snoring decreased. Snoring is a common condition characterized by the production of sound during sleep due to the vibration of respiratory structures. When throat and tongue muscles relax during sleep, the airway narrows, and airflow is partially blocked leading to vibration of the tissues in the throat. The higher snoring index in the non-sleep bruxism group might be understood that these patients had lower muscle tone with higher relaxation and vibration of tissues. Furthermore, increased muscle tone showed a significantly negative correlation to mean duration of desaturation. The significantly increased muscle tone might be an indicator that sleep bruxism could be attributed a protective role in sleep apnea. This protective role to maintain breathing patency and attenuate the severity and occurrence of obstructive sleep apnea (OSA) was already suggested by some authors [1, 9, 26]. On the other side, higher ODI and heart rate, pulse and respiratory related arousals could be estimated as cortical and autonomic activations related to an increase in sympathetic activity. SB could be an autonomic response to restore oxygen saturation. The literature reported that sleep bruxism episodes are strongly associated with arousal activity and therefore with sleep stage shift. But these shifts are transient and macrostructural level is not disturbed [42]. Our results confirmed that sleep bruxism does not affect sleep duration and efficiency. There were no differences in total sleep time and sleep efficiency, thus sleep bruxism patients slept a bit longer and sleep efficiency was a bit higher. A negative impact on sleep macrostructure can therefore be negated.

Based on the literature, sleep bruxism is highly correlated to painful and non-complaint jaw muscle symptoms. Thymi et al. [18] reported that sleep bruxism is not only related to painful but also to non-complaint jaw muscle symptoms. They concluded that there is an importance for future sleep bruxism studies including a comprehensive assessment of not only pain, but also non-complaint muscle symptoms. Therefore, our study investigated painful and non-complaint oral health related symptoms and clinical signs. The DC/TMD protocol [37], considered the current gold standard for researching Temporomandibular Disorders and its assessment, was employed to differentiate between painful symptoms and unreported discomfort. This protocol identified painful muscle symptoms including myalgia, myofascial pain, headache attributed with TMD and positive muscle palpation not mentioned initially by the patient. Probable sleep bruxism patients differ significantly in anamnestic and clinical oral health related symptoms compared to non-sleep bruxism patients. Morning jaw muscle pain had the highest prevalence and was reported by 51.3% of probable sleep bruxers, while non-painful symptoms such as muscle tiredness, muscle stiffness and limited jaw opening upon awakening showed also significantly higher values compared to the non-sleep bruxism group. Limitations in mastication, jaw mobility, and verbal and emotional expression measured with the Jaw Functional Limitation Scale (JFLS-20) [36] showed significantly higher values among probable sleep bruxism patients. The logistic regression model showed a significant contribution of JFLS-20 score to the risk of sleep bruxism.

Therefore, the predictability of sleep bruxism with high scores of unspecific orofacial symptoms like difficulties of chewing hard or soft food is higher in these patients. The clinical examination showed the highest prevalence in non-complaint positive masseter muscle palpation diagnosis in both groups, but with significant higher prevalence in sleep bruxism patients. The same outcome was noted for the painful muscle TMD diagnoses which showed a significantly higher prevalence in probable sleep bruxism patients. Multiple studies have documented connections between painful and non-painful muscle symptoms, Temporomandibular Disorders (TMD) and sleep bruxism [18, 22, 32, 43].

Because of the substantial occurrence of OSA in the general population and its strong correlation with SB, this research emphasized the significance of educating both physicians and dentists to screen for these conditions. Dentists therefore play a significant role to detect the orofacial symptoms in patients with obstructive sleep apnea (OSA). If sleep bruxism is diagnosed clinically, these patients should be screened for sleep disorder breathing with appropriate and validated screening tools for OSA. The Epworth Sleepiness Scale (ESS) [44] and the STOP Bang questionnaire [45] are reliable and in daily practice easy-to-use screening tools for sleep breathing disorders, as they test for typical OSA-related symptoms such as daytime sleepiness, snoring, tiredness, observed apnea, high blood pressure, BMI, age, neck circumference, and male gender. Currently, in dentistry the awareness of an association between SB and OSA is low, thus there is a future need for clarification and interdisciplinary approach for undiagnosed OSA patients. According to Alessandri-Bonetti et al. [46] the prevalence of TMD signs and symptoms were significantly higher in untreated OSA patients when compared to controls. A reduction in signs and symptoms of both TMD and OSA can be expected with OSA treatment [47]. Based on signs of severe tooth wear, unspecific orofacial symptoms such as specific TMD symptoms and positive sleep questionnaires, dentists could be the first to diagnose patients with OSA in an interdisciplinary approach. Currently in daily clinical practice, undiagnosed OSA patients with SB are often treated with intermaxillary splints, which could lead to worsening OSA symptoms and their comorbidities like heart diseases, diabetes mellitus and depression [48]. Furthermore, early diagnosis of untreated OSA patients in dentistry plays a crucial role avoiding comorbidities and reducing economic burden in medicine. Hence, collaborative care involving physicians, otolaryngologists, and dentists is vital for the overall oral and medical well-being of these patients.

## Conclusions

- More than 30% of patients with obstructive sleep apnea show sleep bruxism.
- Electromyographic muscle tone were significantly higher in sleep bruxism patients in all sleep stages and can used as a predictable value for sleep bruxism risk.
- Sleep bruxism in OSA patients is not only a dental problem. Medical signs are shown in higher AHI, RDI, ODI, AI, HI values, significant higher number of hypopnea, and higher sympathetic activities in SB patients. Therefore, these patients should be screened by dentists with anamnestic sleep questionnaires regarding sleep breathing disorders.
- Consequently, interdisciplinary treatment between medical and dental clinicians is important for greater understanding the etiology and appropriate therapeutic intervention in these patients.

## Data Availability

All data generated or analyzed during this study are included in this article. Further inquiries can be directed to the corresponding author.

## Abbreviations

OSA: Obstructive sleep apnea
SB: Sleep bruxism
NSB: No sleep bruxism
PSG: Polysomnography
PG: Polygraphy
TMD: Temporomandibular disorder
DC/TMD: Diagnostic Criteria of Temporomandibular Disorders
JFLS-20: Jaw Functional Limited Scale (20 items)
RMMA: Rhythmic masticatory muscular activity
N1: Sleep stage 1
N2: Sleep stage 2
N3: Sleep stage 3
REM: Rapid eye movement sleep stage
TST: Total sleep time
SE: Sleep efficiency
AHI: Apnea hypopnea index
AI: Apnea index
HI: Hypopnea index
RDI: Respiratory disturbance index
ODI: Oxygen desaturation index
SI: Snoring index
